# Identification and Validation of Prognostic Model for Pancreatic Ductal Adenocarcinoma Based on Necroptosis-Related Genes

**DOI:** 10.3389/fgene.2022.919638

**Published:** 2022-06-16

**Authors:** Haoran Xie, Jingxian Xu, Zhiwen Xie, Ni Xie, Jiawei Lu, Lanting Yu, Baiwen Li, Li Cheng

**Affiliations:** ^1^ Department of Gastroenterology, Shanghai General Hospital, Shanghai Jiao Tong University School of Medicine, Shanghai, China; ^2^ Shanghai Key Laboratory of Pancreatic Diseases, Shanghai General Hospital, Shanghai Jiao Tong University School of Medicine, Shanghai, China; ^3^ Department of Urology, Shanghai General Hospital, Shanghai Jiao Tong University School of Medicine, Shanghai, China; ^4^ Department of International Medical Care Center, Shanghai General Hospital, Shanghai Jiao Tong University School of Medicine, Shanghai, China

**Keywords:** necroptosis, pancreatic ductal adenocarcinoma, prognostic model, risk score, immune infiltration

## Abstract

**Background:** Pancreatic ductal adenocarcinoma (PDAC) is one of the most malignant tumors with a poor prognosis. Recently, necroptosis has been reported to participate in the progression of multiple tumors. However, few studies have revealed the relationship between necroptosis and PDAC, and the role of necroptosis in PDAC has not yet been clarified.

**Methods:** The mRNA expression data and corresponding clinical information of PDAC patients were downloaded from the TCGA and GEO databases. The necroptosis-related genes (NRGs) were obtained from the CUSABIO website. Consensus clustering was performed to divide PDAC patients into two clusters. Univariate and LASSO Cox regression analyses were applied to screen the NRGs related to prognosis to construct the prognostic model. The predictive value of the prognostic model was evaluated by Kaplan-Meier survival analysis and ROC curve. Univariate and multivariate Cox regression analyses were used to evaluate whether the risk score could be used as an independent predictor of PDAC prognosis. Gene Ontology (GO), Kyoto Encyclopedia of Genes and Genomes (KEGG) and single-sample gene set enrichment analysis (ssGSEA) were used for functional enrichment analysis. Finally, using qRT-PCR examined NRGs mRNA expression *in vitro*.

**Results:** Based on the TCGA database, a total of 22 differential expressed NRGs were identified, among which eight NRGs (CAPN2, CHMP4C, PLA2G4F, PYGB, BCL2, JAK3, PLA2G4C and STAT4) that may be related to prognosis were screened by univariate Cox regression analysis. And CAPN2, CHMP4C, PLA2G4C and STAT4 were further selected to construct the prognostic model. Kaplan-Meier survival analysis and ROC curve showed that there was a significant correlation between the risk model and prognosis. Univariate and multivariate Cox regression analyses showed that the risk score of the prognostic model could be used as an independent predictor. The model efficacy was further demonstrated in the GEO cohort. Functional analysis revealed that there were significant differences in immune status between high and low-risk groups. Finally, the qRT-PCR results revealed a similar dysregulation of NRGs in PDAC cell lines.

**Conclusion:** This study successfully constructed and verified a prognostic model based on NRGs, which has a good predictive value for the prognosis of PDAC patients.

## Introduction

Pancreatic ductal adenocarcinoma (PDAC) is the most malignant tumor of the digestive tract (Caldwell et al., 2021). Although the incidence of PDAC is not high, the mortality rate is high, making it the fourth leading cause of cancer-related deaths (Siegel et al., 2021). PDAC developed so rapidly that most patients were diagnosed only when the disease has progressed to an advanced stage or distant metastasis. Even if it can be treated by surgical resection, it is easy to relapse (Avula et al., 2020; Lotfi et al., 2021). Therefore, the identification of potential molecular mechanisms and therapeutic targets is very important for the early diagnosis, treatment and prognosis of pancreatic cancer.

Cell death is one of the most concerned fields in tumor research. Necroptosis is one of the regulated forms of cell death unmediated by caspases (Gong et al., 2019). The morphological characteristics of necroptosis showed swelling of cells, cytoplasmic vacuolization and explosive rupture of cell membrane (Liu et al., 2021b). In addition, necrotic cells caused the inflammatory response of surrounding cells and activated the body’s immune response by releasing its contents. Therefore, necroptosis plays an important role in inflammatory diseases, including IBD, acute renal injury and inflammatory response syndrome, etc., (Grootjans et al., 2017; Tonnus et al., 2019). Recent studies have shown that necroptosis also plays an important role in tumorigenesis and anticancer. For example, Yu et al. (2022) found that SMYD2 could target RIPK1 to repress necroptosis and promote the growth of colon cancer. Li et al. (2021) revealed that necroptosis-related genes ALOX15, BCL2, IFNA1, PYGL and TLR3 may be closely related to the poor prognosis of prostate cancer. However, the role and significance of necroptosis in PDAC remain unclear.

Hence, the purpose of this study is to analyze the differential expression of necroptosis-related genes (NRGs) in normal tissues and PDAC tissues, and to construct a risk prognosis model based on NRGs by univariate and least absolute shrinkage and selection operator (LASSO) Cox regression analyses, which could provide accurate prognosis prediction for PDAC patients.

## Material and Methods

### Data Collection

The mRNA expression data of 182 samples (4 normal tissues and 178 PDAC tissues) and corresponding clinical information were downloaded from The Cancer Genome Atlas (TCGA) website (https://portal.gdc.cancer.gov/repository) to be used as training cohort to establish the prognostic model. GSE57495 dataset, containing the mRNA expression profile and related clinical features of 63 PDAC patients, was downloaded from NCBI Gene Expression Omnibus (GEO) (https://www.ncbi.nlm.nih.gov/geo/) to be used as test cohort to validate the model. In addition, 147 NRGs obtained from the CUSABIO website (http://www.cusabio.cn/pathway/Necroptosis.html) were listed in Supplementary Table S1.

### Identification of Differentially Expressed Necroptosis-Related Genes

The “limma” R package was used to analyze the differential expression of NRGs between the normal tissues and PDAC tissues in the TCGA cohort. *p*-value < 0.05 was set to identify the differentially expressed NRGs, visualized by the heatmap. The Protein-Protein Interaction (PPI) network of the differentially expressed NRGs was constructed by the STRING database (http://string-db.org/).

### Consensus Clustering Analysis

Based on the survival time and status of PDAC patients in the TCGA cohort, the “ConsensusClusterPlus” R package was used to carry out the consensus clustering analysis. Due to the randomness of k-means clustering analysis, the clustering index “k” was increased from 2 to 10 to determine the clustering index with the least interference and the largest difference between clusters. The survival curve was conducted using the “survival” and “survminer” R packages. Then, NRGs were further differentially analyzed based on different clusters using the“limma” R package (|Log2(FC)|>0.585 and FDR<0.05). And the heatmap was plotted to show the relationship between NRGs and clinical features.

### Construction and Validation of Prognostic Model based on Necroptosis-Related Genes

The univariate Cox regression analysis was utilized to evaluate the prognostic value of NRGs. To avoid omission, the threshold *p*-value was set to 0.25 and 8 genes were selected for subsequent study. Then, LASSO Cox regression analysis, which could reduce the risk of overfitting, was used to establish the prognostic model. The optimum λ was chosen by the minimum criteria of the penalized maximum likelihood estimator in a 10-fold cross-validation. In this model, the risk score of each PDAC patient in TCGA and GEO cohorts was calculated by multiplying the coefficients of the gene and the expression of genes. The principal component analysis (PCA) was performed by using the “Rtsne” R package. Subsequently, these patients were divided into two groups (high-risk and low-risk groups) according to the median risk score. The predictive accuracy of this model was evaluated by survival analysis and time-dependent ROC curve, which were performed by the “Survminer” R package and the “timeROC” R package, respectively. Finally, we conducted the univariate and multivariate Cox regression analyses to assess the prognostic value of the risk model and whether the model could be served as an independent prognostic predicting factor.

### Functional Enrichment Analysis

Based on the risk subgroups, the differentially expressed genes (DEGs) with log2(FC) > 1 or < −1 and FDR <0.05 in the TCGA cohort were obtained. Then, the “clusterProfiler” R package was subjected to the Gene Ontology (GO) and Kyoto Encyclopedia of Genes and Genomes (KEGG) enrichment analyses. To assess the status of 16 kinds of immune cells and 13 kinds of immune-linked functions, we performed the single-sample gene set enrichment analysis (ssGSEA) to calculate the immune score using the “gsva” R packet.

### Cell Culture

The human normal pancreatic ductal epithelial cell line (HPDE6-c7) and human pancreatic cancer cell line (PANC-1) were cultured in DMEM (Gibco) supplemented with 10% fetal bovine serum (FBS, Gibco) and 1% penicillin and streptomycin (Gibco). The human pancreatic cancer cell line (AsPC-1) was maintained in RPMI-1640 medium (Gibco) containing 10% FBS and 1% penicillin and streptomycin. All cell lines were purchased from the Type Culture Collection of the Chinese Academy of Science (Shanghai, China). All cell lines were cultured in a humidified incubator at 37 °C with 5% CO_2_.

### Examination of the Expression of Four Necroptosis-Related Genes

Total RNA of cells was extracted using RNAiso Plus Reagent (Takara) and was reverse-transcribed to cDNA *via* the PrimeScriptTM RT reagent kit (Takara). Then, SYBR Premix Ex Taq (Takara) was used to perform qRT–PCR assays. GAPDH was used as the internal control, and the relative expression levels were calculated by the 2^−ΔΔCt^ method. Primers sequences are shown in Supplementary Table S2.

The protein levels of NRGs were examined by immunohistochemical results obtained from HPA (Human Protein Atlas) database (https://www.proteinatlas.org/). The relationship between NRGs expression and overall survival was obtained from the GEPIA database.

### Statistical Analysis

Statistical analysis of all data was carried out using R version 4.0.4 in this study. Statistical significance was defined as *p* < 0.05.

## Results

### Dataset Characteristics and Identification of Necroptosis-Related Genes

**FIGURE 1:**
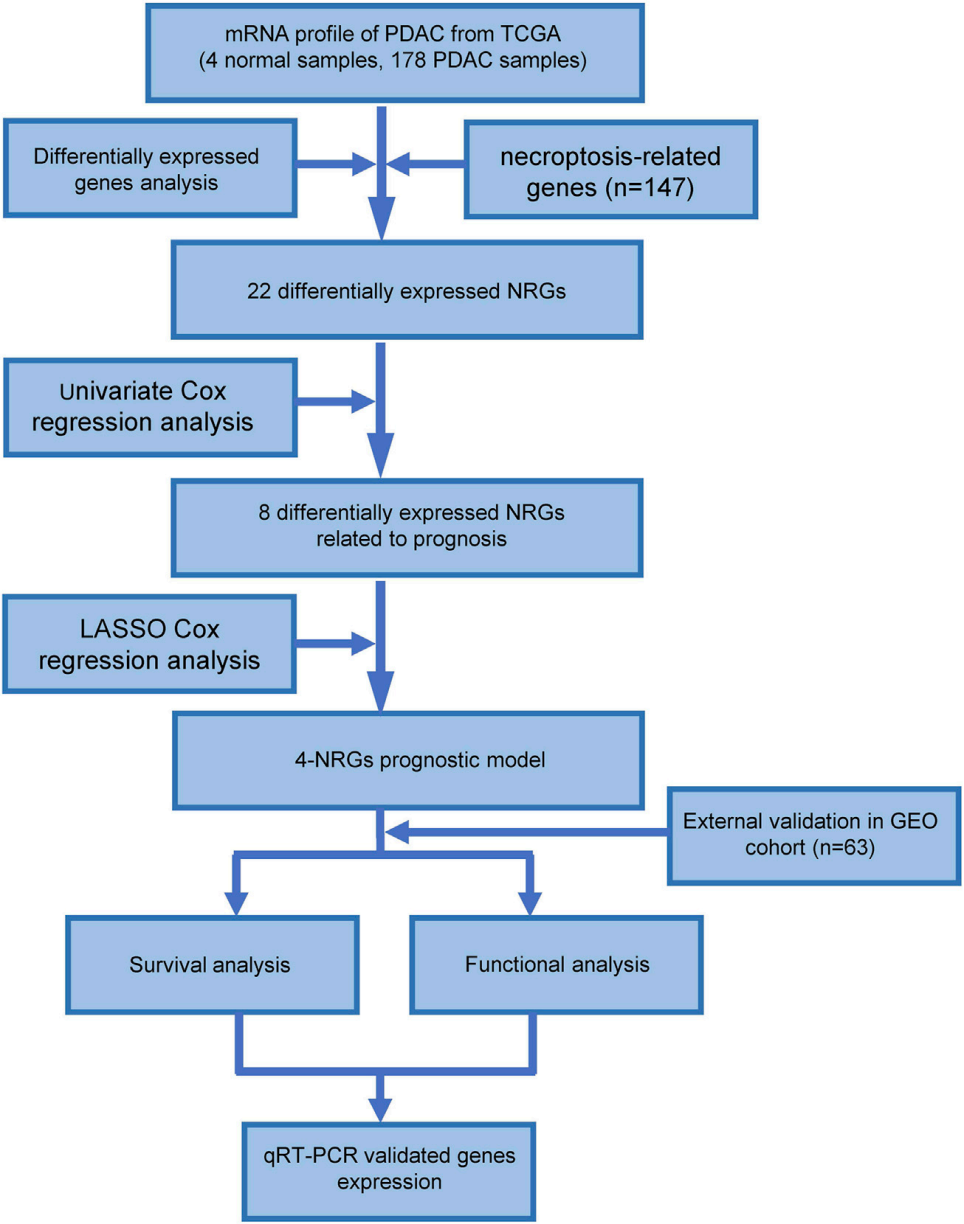
Workflow of the study.

The overall design of the study was shown in Figure 1. In the TCGA dataset, 178 tumor samples and 4 normal samples were included. In the GEO dataset, 63 tumor samples were subjected to external validation. The detailed demographic and clinical characteristics were shown in Supplementary Table S3.

**FIGURE 2:**
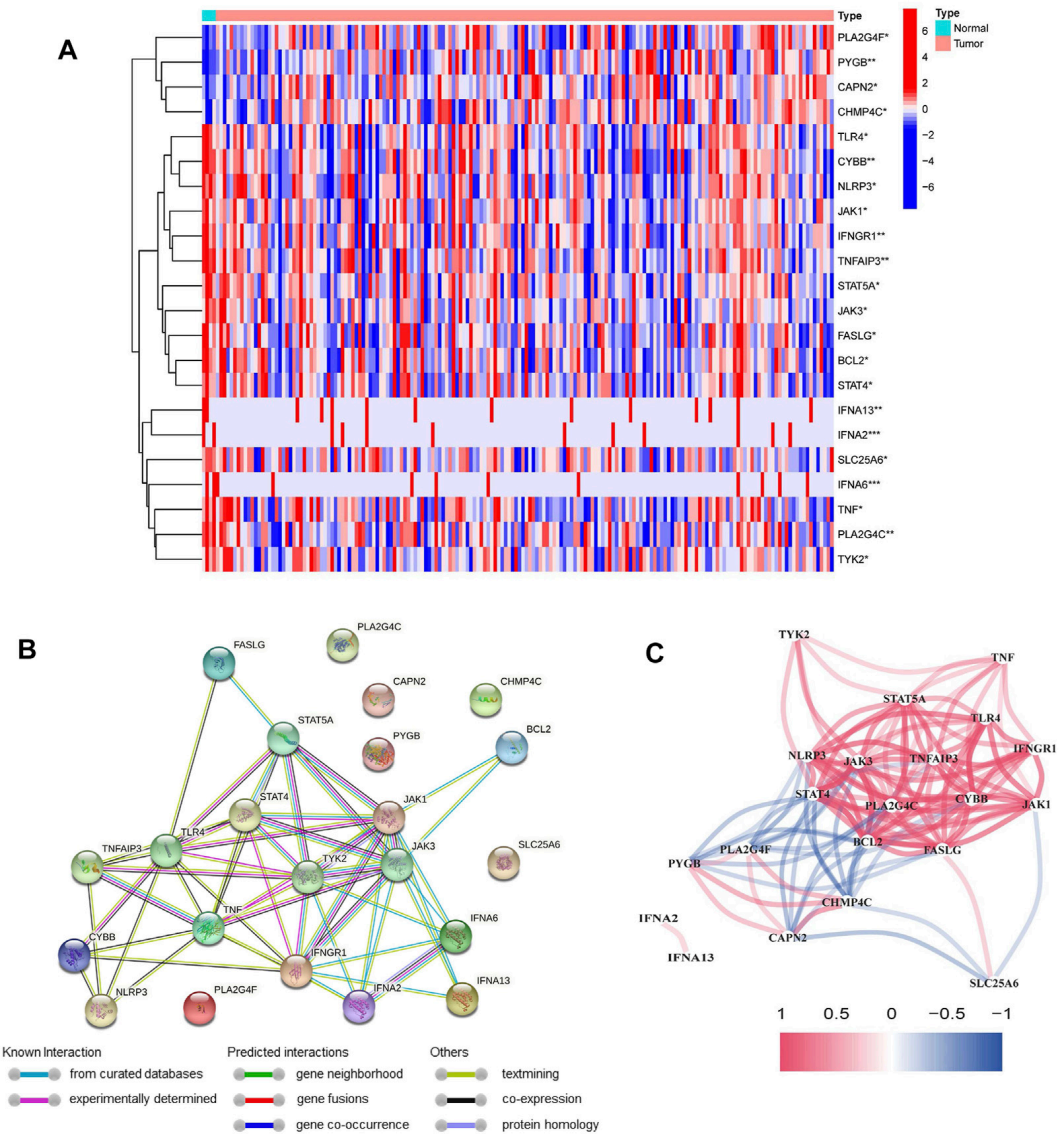
Identification of differentially expressed NRGs in the TCGA cohort. **(A)** Heatmap of differentially expressed NRGs between normal samples and PDAC samples (*p < 0.05, **p < 0.01, ***p < 0.001). **(B)** The PPI network of differentially expressed NRGs. **(C)** The correlation network of differentially expressed NRGs (red line: positive correlation; blue line: negative correlation).

131 out of 147 NRGs were used to detect the differential expression between PDAC and normal tissues (Supplementary Table S4). We identified 22 differentially expressed NRGs, among which 4 genes (CAPN, CHMP4C, PYGB and PLA2G4F) were upregulated and 18 genes (IFNA6, IFNA2, IFNA13, BCL2, TNF, CYBB, FASLG, JAK3, STAT4, TNFAIP3, PLA2G4C, TLR4, NLRP3, IFNGR1, STAT5A, TYK2, JAK1 and SLC25A6) were downregulated in PDAC tissues (Figure 2A). The PPI network of NRGs established with the minimum required interaction score > 0.4 was shown in Figure 2B. The correlation network of NRGs was illustrated in Figure 2C.

### Tumor Classification Based on the Necroptosis-Related Genes

**FIGURE 3:**
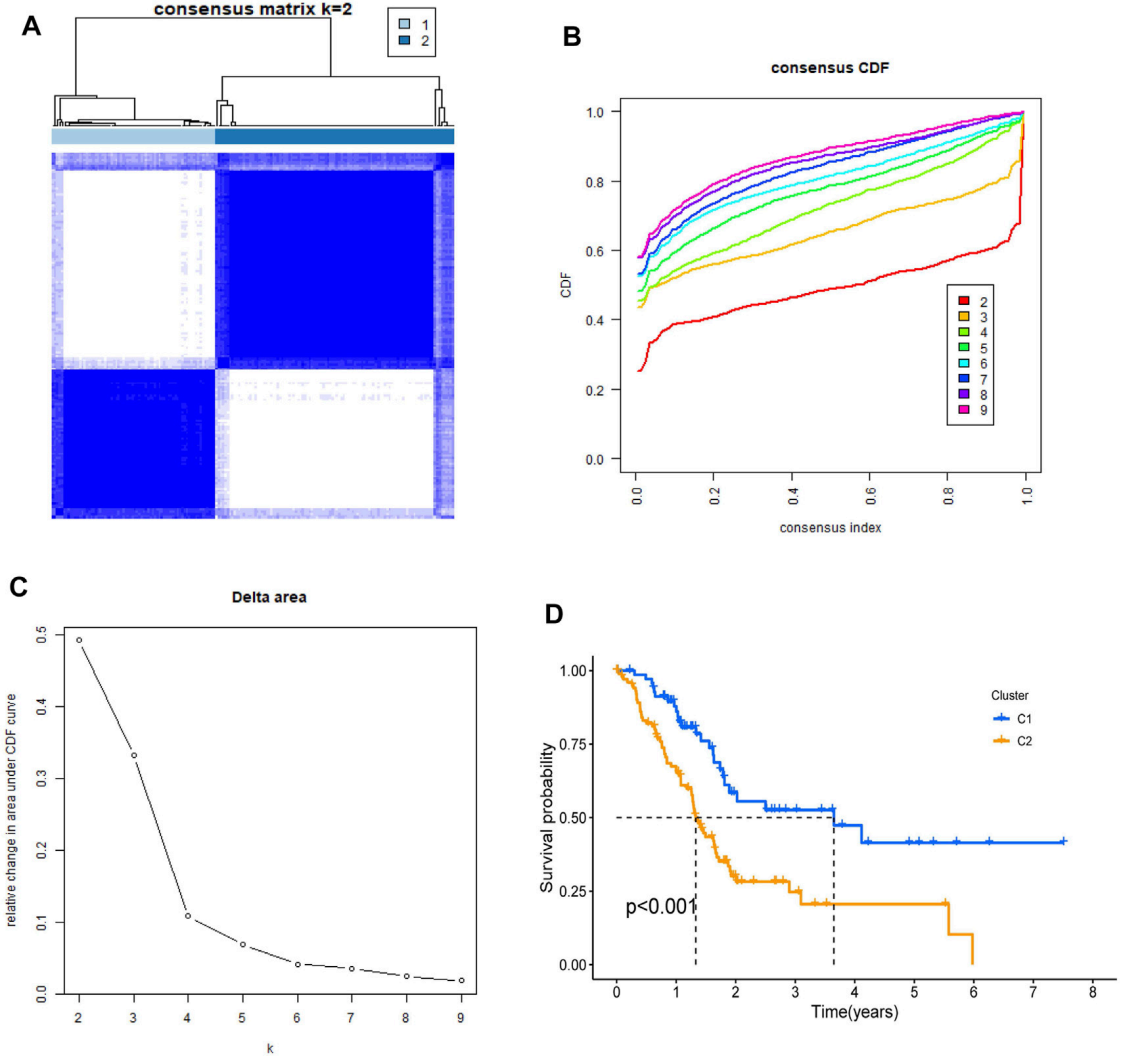
Identification of tumor classification based on the NRGs. **(A)** PDAC patients were classified into two clusters by NRGs-based consensus clustering analysis (k = 2). **(B,C)** Cumulative Distribution function (CDF) consistent clustering model with k from 2 to 9. **(D)** Kaplan–Meier survival analysis in two clusters.

To investigate the relationship between the 22 NRGs and PDAC subtypes, the consensus clustering analysis was performed in the TCGA cohort. By increasing the clustering index “k” from 2 to 10, we found that when k = 2, intragroup connections were the highest and intergroup connections were the lowest, showing that the PDAC patients could be grouped into two clusters (Figures 3A–C and Supplementary Figure S1). In addition, survival analysis was conducted and showed that compared to cluster 1, cluster 2 had a worse prognosis (*p* < 0.001, Figure 3D).

**FIGURE 4:**
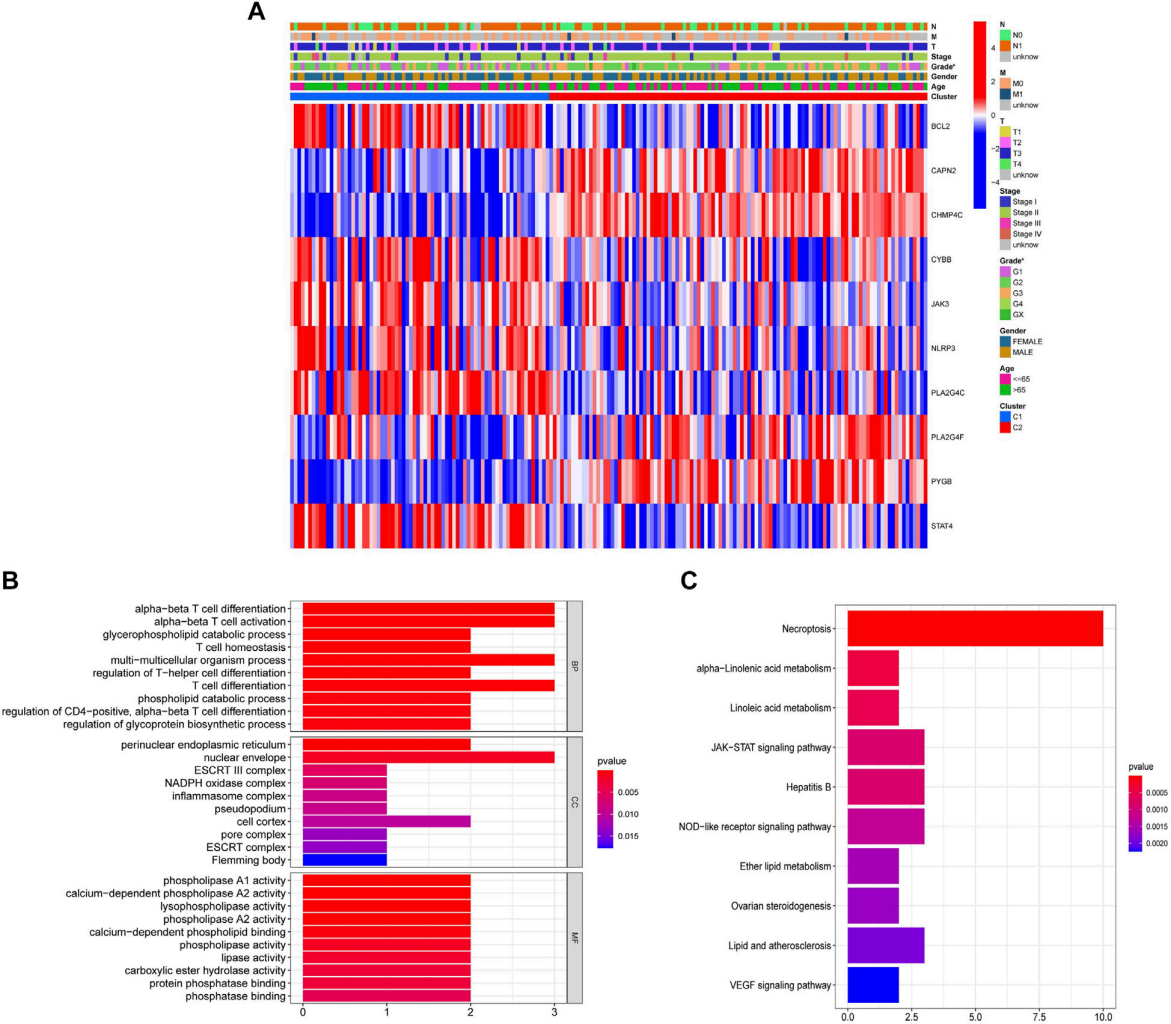
Identification of the differential expression of 22 NRGs between cluster 1 and cluster 2. **(A)** Heatmap with the correlation between 10 differentially expressed NRGs in two clusters and clinical characteristics (*p < 0.05). **(B)** GO analysis of 10 differentially expressed NRGs. **(C)** KEGG analysis of 10 differentially expressed NRGs.

Subsequently, we identified 10 of the 22 NRGs with different expressions between cluster 1 and cluster 2. The heatmap showed that there are significant differences in NRGs expression and Grade (Figure 4A). These 10 NRGs were then subjected to GO and KEGG analyses to uncover their biological function. GO analysis suggested that NRGs were related to T cell differentiation and activation that was associated with the anti-tumor effect and multiple enzyme activities that could regulate necroptosis (Figure 4B). Moreover, KEGG analysis revealed that NRGs were predominantly enriched in necroptosis, as well as related metabolic pathways and the JAK-STAT signaling pathway (Figure 4C).

### Construction of the Necroptosis-Related Genes-Based Independent Prognostic Risk Model in The Cancer Genome Atlas Cohort

**FIGURE 5:**
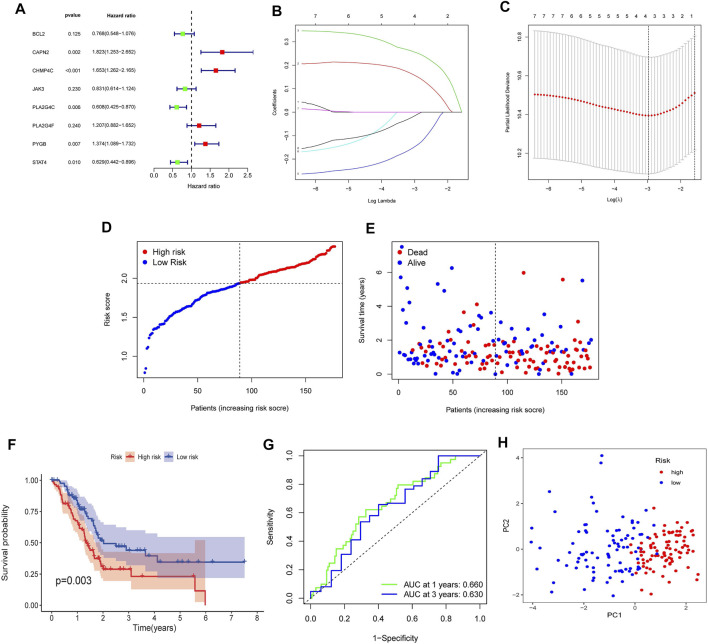
Construction of the NRGs-based prognostic risk model in the TCGA Cohort. **(A)** Univariate Cox regression analysis for 10 NRGs. **(B)** LASSO Cox regression analysis of 8 NRGs. **(C)** Cross validation method to select optimal genes. **(D)** Distribution of risk score. **(E)** The survival status and survival time distribution. **(F)** Kaplan-Meier survival analysis between the high-risk group and the low-risk group. **(G)** Time-dependent ROC curve to evaluate the efficiency of the model. **(H)** Results of PCA analysis.

To verify the prognostic significance of the ten NRGs mentioned above, we first conducted univariate Cox regression analysis. When we set 0.25 as the *p*-value, a total of eight genes were selected (Figure 5A). Among which CAPN2, CHMP4C, PLA2G4F and PYGB were deemed as high-risk genes (HR > 1), while BCL2, JAK3, PLA2G4C and STAT4 were deemed as low-risk genes (HR < 1). Subsequently, the LASSO Cox regression analysis was implemented to construct the risk model. Four genes were selected based on the optimum penalty parameter (λ) value (Figures 5B,C). The risk score was obtained calculated by the equation: risk score = (CAPN2 × 0.1549) + (CHMP4C × 0.2546) + (PLA2G4C × −0.1449) + (STAT4 × −0.0136). Based on the median risk score, patients in the TCGA cohort were separated into high-risk group and low-risk group (Figure 5D). Patients with a high score had more deaths and shorter overall survival (OS) (Figure 5E). Furthermore, Kaplan–Meier survival analysis was performed and indicated that patients in the high-risk group had worse OS rates (*p* = 0.003, Figure 5F). Then time-dependent receiver operating characteristics (ROC) analysis was used to evaluate the perfection of the prognostic model (Figure 5G). The area under the curve (AUC) in 1- and 3 years were 0.660 and 0.630, respectively. Finally, the principal component analysis (PCA) indicated that patients in high- and low-risk groups could be well divided into two groups (Figure 5H).

### Identification of the Necroptosis-Related Genes Risk Model as an Independent Prognostic Predicting Factor for Overall Survival

**FIGURE 6:**
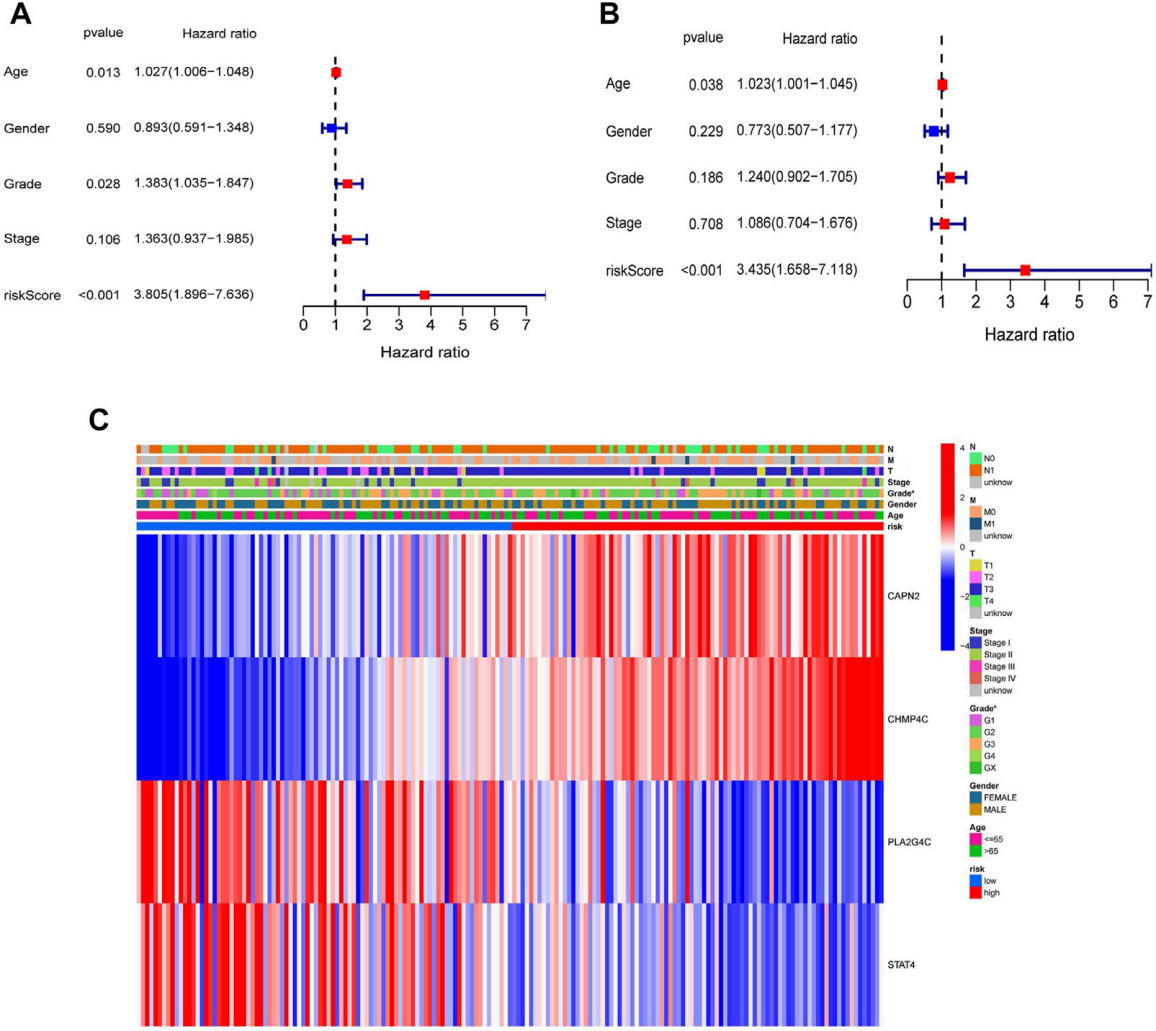
Univariate and multivariate Cox regression analyses for the risk score and clinical features in the TCGA cohort. **(A)** Univariate Cox regression analysis. **(B)** Multivariate Cox regression analysis. **(C)** Heatmap with the correlation between the expression of four NRGs in different risk groups and clinical features (*p < 0.05).

To assess whether the NRGs risk model could be used as an independent prognostic factor, we performed the univariate and multivariate Cox regression analyses. The results of the univariate Cox regression analysis revealed that the Age (HR = 1.027, 95% CI = 1.006–1.048, *p* = 0.013), Grade (HR = 1.383, 95% CI = 1.035–1.847, *p* = 0.028) and riskScore (HR = 3.805, 95% CI = 1.896–7.636, *p* < 0.001) were remarkably related to OS (Figure 6A). The multivariate Cox regression analysis further demonstrated that the Age (HR = 1.023, 95% CI = 1.001–1.045, *p* = 0.038) and riskScore (HR = 3.435, 95% CI = 1.658–7.118, *p* < 0.001) were the independent predicting factors (Figure 6B). Moreover, the heatmap was plotted to reveal the relationship between the expression levels of four genes in different risk groups and clinical characteristics (Figure 6C). The above results indicated that the risk model was the independent prognostic factor for PDAC.

### External Validation of the Necroptosis-Related Genes-Based Prognostic Risk Model in Gene Expression Omnibus Cohort

**FIGURE 7:**
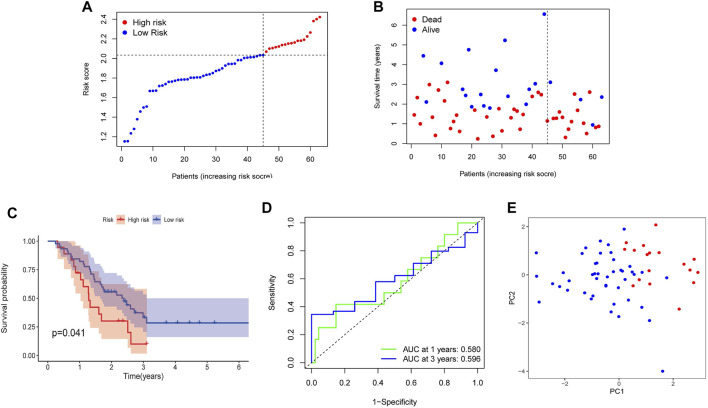
Validation of the prognostic risk model in the GEO cohort. **(A)** Distribution of risk score. **(B)** The survival status and survival time distribution. **(C)** Kaplan-Meier survival analysis between the high-risk group and the low-risk group. **(D)** Time-dependent ROC curve to verify the efficiency of the model. **(E)** Results of PCA analysis.

For further verifying the efficacy of four NRGs-based prognostic model, the GEO cohort (GSE57495) containing 63 PDAC patients was used as a validation cohort. According to the risk score, 63 patients were divided into the high-risk (*n* = 18) and low-risk groups (*n* = 45) (Figure 7A). Patients in the high-risk group were likely to have higher death rates and shorter OS (Figure 7B). And the Kaplan–Meier survival analysis also displayed that the high-risk group had a worse survival probability than those in the low-risk group (*p* = 0.041, Figure 7C). Furthermore, the AUC in 1 and 3 years were 0.580 and 0.596 (Figure 7D). Finally, the PCA analysis result showed a clear separation between the two subgroups (Figure 7E). The results of the above analysis indicated that the model in our study had a pretty good predictive efficacy. However, the univariate and multivariate Cox regression analyses in the GEO cohort showed there was no significance in risk score and clinical characteristics, which may be caused by small sample size (Supplementary Figure S2).

### Functional Enrichment Analysis of DEGs Based on the Risk Score

**FIGURE 8:**
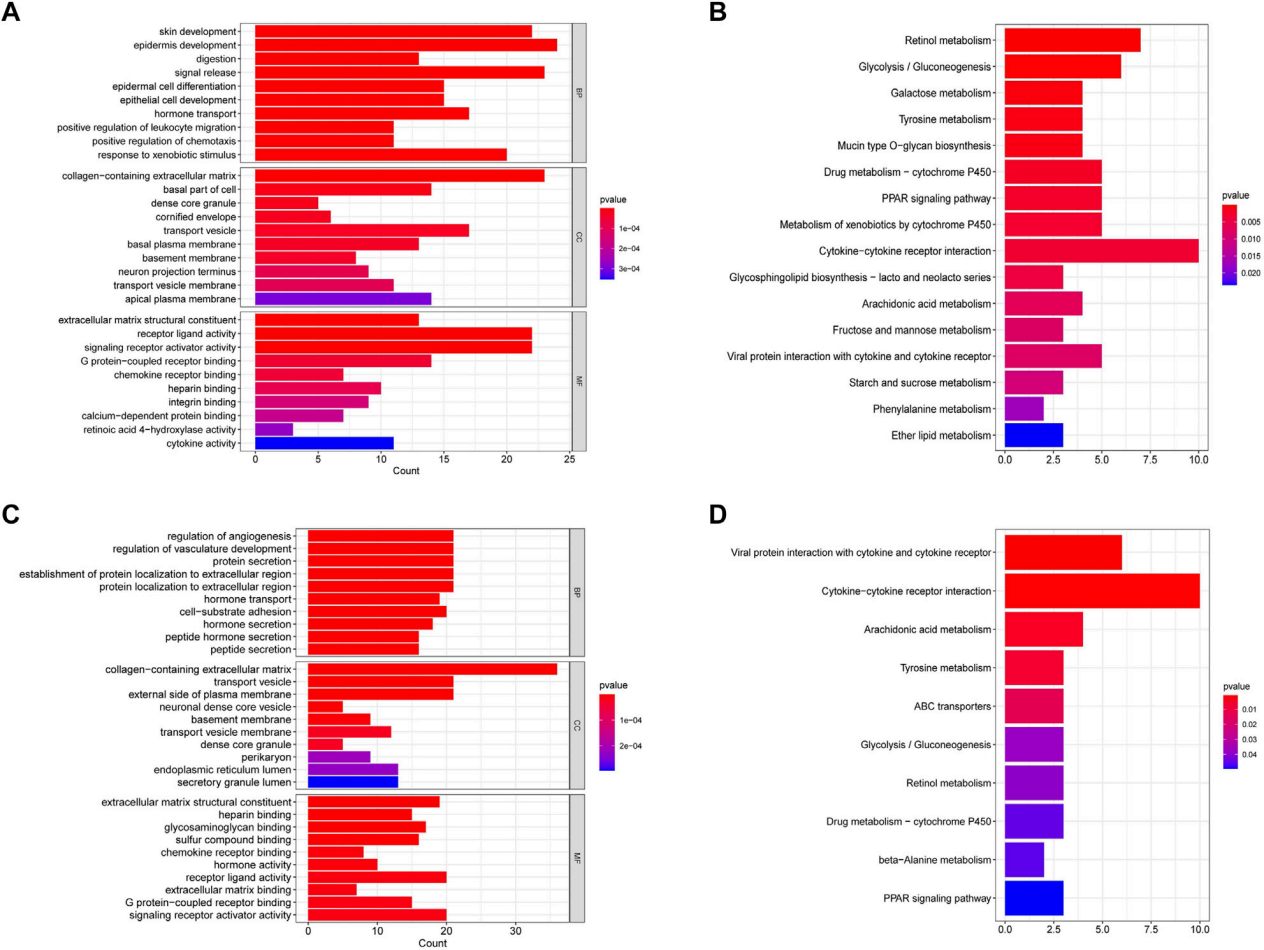
Functional enrichment analysis of differentially expressed genes in the TCGA and GEO cohorts. **(A)** GO analysis of differentially expressed genes in the TCGA cohort. **(B)** KEGG pathway analysis of differentially expressed genes in the TCGA cohort. **(C)** GO analysis of differentially expressed genes in the GEO cohort. **(D)** KEGG pathway analysis of differentially expressed genes in the GEO cohort.

To explore the function of genes in different risk subgroups, we identified 259 DEGs in the TCGA cohort (Supplementary Table S5) and 257 DEGs in the GEO cohort (Supplementary Table S6). Subsequently, these genes were subjected to the GO and KEGG pathway analyses. GO analysis showed that the DEGs in TCGA and GEO cohorts were significantly relevant to signal release (Biological Process, BP), collagen−containing extracellular matrix (Cellular Component, CC) and receptor ligand activity (Molecular Function, MF) (Figures 8A,C). Moreover, KEGG analysis indicated that the DEGs were predominantly enriched in cytokine−cytokine receptor interaction (Figures 8B,D).

### Immune Status and Necroptosis-Related Risk Score

**FIGURE 9:**
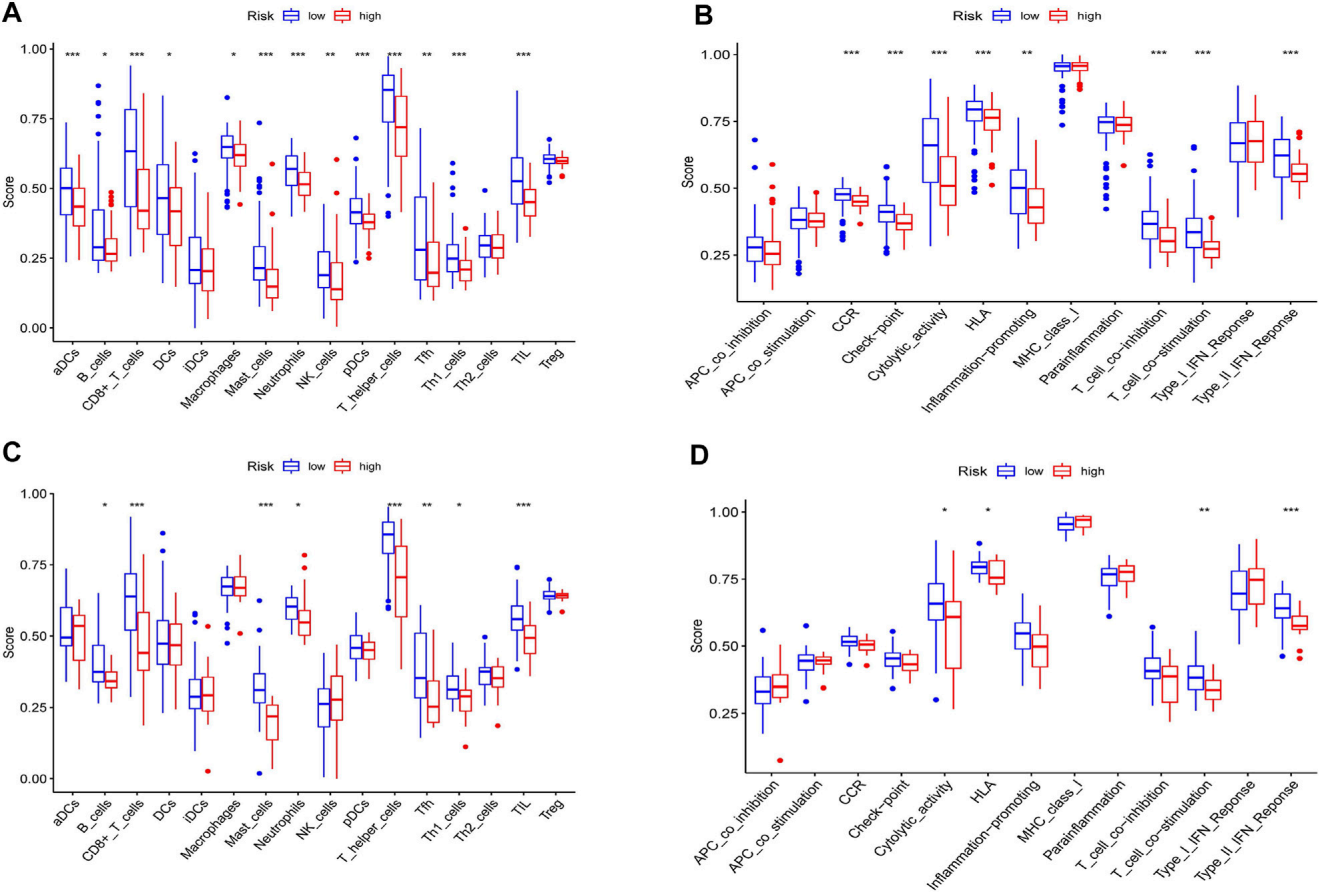
Immune status analysis between high and low-risk groups in the TCGA and GEO cohorts. **(A)** Comparisons of the score of 16 immune cells between different risk groups in the TCGA cohort. **(B)** Comparisons of the score of 13 immune functions between different risk groups in the TCGA cohort. **(C)** Comparisons of the score of 16 immune cells between different risk groups in the GEO cohort. **(D)** Comparisons of the score of 13 immune functions between different risk groups in the GEO cohort. *p < 0.05, **p < 0.01, ***p < 0.001.

To investigate the relationship between the immune status and the risk score in TCGA and GEO cohorts, the ssGSEA was used to analyze the differences in 16 kinds of immune cell infiltrations and 13 kinds of immune-linked functions. We found that in the TCGA cohort the high-risk group had the lower infiltration degrees of activated dendritic cells (aDCs), CD8^+^ T cells, T helper (Th) cells, T follicular helper (Tfh) cell, Th1 cells, mast cells, neutrophils, natural killer (NK) cells, plasmacytoid DC (pDC), tumor-infiltrating lymphocyte (TIL) compared to the low-risk group (*p* < 0.05, Figure 9A). Moreover, the high-risk group also displayed inhibition in immune-linked functions, involving CCR, Check−point, Cytolytic activity, HLA, Inflammation promotion, T cell co-inhibition, T cell co-stimulation and Type II IFN Response (*p* < 0.05, Figure 9B). In the GEO cohort, we got similar results that the immune cell infiltrations and the immune-linked functions were decreased (Figures 9C,D). In conclusion, our result indicated that the immune state was significantly related to the risk score and patients with high-risk scores had relatively inactivated immune status compared to those with low-risk scores.

### Detection of the Expression of Four Necroptosis-Related Genes

**FIGURE 10:**
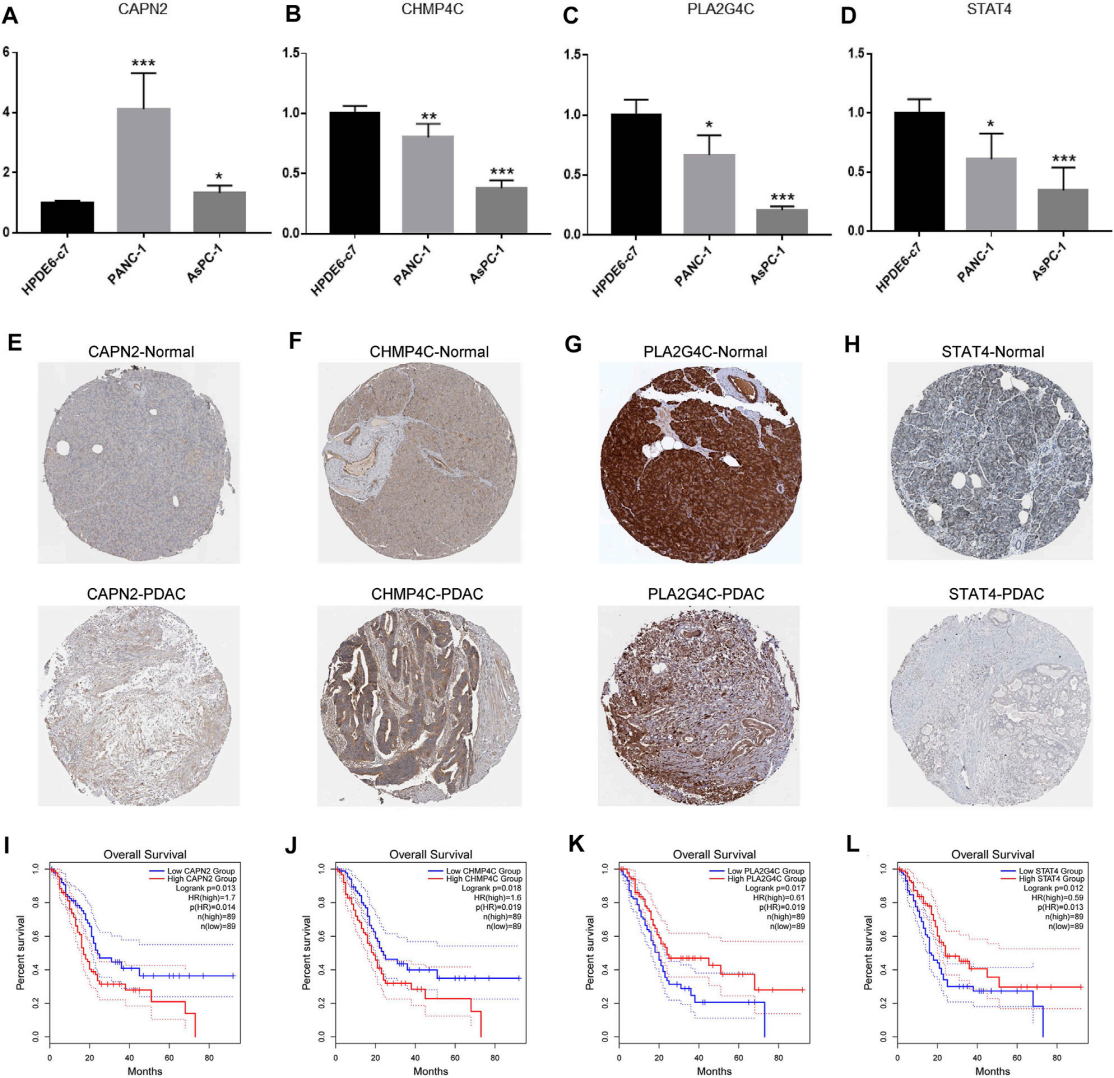
Detection of expression levels of the four NRGs in PDAC. **(A–D)** The mRNA expression of CAPN2, CHMP4C, PLA2G4C and STAT4 in normal pancreatic ductal epithelial cell line (HPDE6-c7) and human pancreatic cancer cell lines (PANC-1, AsPC-1). **(E–H)** Immunohistochemistry showed the protein levels of CAPN2, CHMP4C, PLA2G4C and STAT4 in normal and PDAC tissues. **(I–L)** Kaplan-Meier curve revealed the relationship between survival time and the expression of CAPN2, CHMP4C, PLA2G4C and STAT4. *p < 0.05, ** *p* < 0.01, ***p < 0.001.

We first detected the mRNA levels of four NRGs in cell lines. As shown in Figures 10A–D, the PDAC cell lines (PANC-1, AsPC-1) had lower mRNA levels of CHMP4C, PLA2G4C and STAT4 than normal pancreatic ductal epithelial cell line (HPDE6-c7). Only CAPN2 had higher mRNA levels in the PDAC cell lines.

Subsequently, we further explored the protein levels of the four NRGs between normal tissues and PDAC tissues and the relationship between NRGs expression and overall survival of PDAC patients by the HPA and GEPIA databases, respectively. The results showed that the PDAC tissues had higher protein levels of CAPN2 and CHMP4C than normal tissues (Figures 10E,F). Furthermore, patients with high expression of CAPN2 and CHMP4C had a shorter survival time (Figures 10I,J). On the contrary, PLA2G4C and STAT4 showed lower protein levels in PDAC tissues (Figures 10G,H), which indicated worse overall survival (Figures 10K,L).

## Discussion

Necroptosis is an important programmed cell death, which is characterized by the activation of MLKL/pMLKL by the RIPK1/RIPK3-mediated phosphorylation signal pathway (Karlowitz and van Wijk 2021; Zhou et al., 2022). Recent studies have found that NRGs play a dual role in both facilitating and suppressing the progression of different tumor cells and could be used as prognostic and therapeutic biomarkers in patients with many types of tumors (Pasparakis and Vandenabeele, 2015; Li et al., 2020). In this study, we downloaded the transcriptome data and corresponding clinical information of PDAC patients from the TCGA database and constructed the NRGs-based independent prognostic risk model by univariate and LASSO Cox regression analyses. The GEO cohort was used for external validation. In addition, this study further explored the role of immune cell infiltration in the tumor microenvironment in pancreatic cancer.

In the current study, we first identified 22 differentially expressed NRGs in normal tissues and PDAC tissues. Consensus clustering analysis is an effective method to identify different subtypes of tumors and survival patterns (Brannon et al., 2010; Wang et al., 2014). Based on differentially expressed NRGs, consensus clustering analysis divides the patients in the TCGA cohort into two clusters. PDAC patients in cluster one had a significantly better prognosis. Remarkably, the previous study has reported that trimethyltin chloride could activate NLRP3 inflammasome to induce necroptosis (Liu et al., 2022). Consistent with previous studies, our study also found NLRP3 was significantly upregulated in cluster one, which may be associated with potential mechanisms of different prognosis in different clusters.

Subsequently, we constructed a risk prognostic model composed of four NRGs (CAPN2, CHMP4C, PLA2G4C, STAT4) by using univariate and LASSO Cox regression analyses. In the prognostic model, the survival time of patients in the high-risk group was significantly less than that in the low-risk group. Further univariate and multivariate Cox regression analyses showed that the risk score of the prognostic model could be used as an independent prognostic factor for patients with PDAC. Genes in this model have been reported in a few studies of tumors. Calpain 2 (CAPN2) is one of the most important members of the calpain family. It has been reported that CAPN2 is involved in the development of a variety of tumors, including liver cancer, gastric cancer and so on (Shen et al., 2012; Liu et al., 2017). In pancreatic cancer, it has been reported that CAPN2 can induce apoptosis of pancreatic cancer cells and is related to poor prognosis (Guo et al., 2020; Szymczak-Pajor et al., 2021). Phospholipase A2 Group IV C(PLA2G4C) encoded a protein that is a calcium-independent membrane binding enzyme with phospholipase A2, lysophospholipase and transacyl activity. Studies have shown that it can regulate tumor cell chemotaxis and invasion by regulating the AKT signal pathway (Tian et al., 2011; Olsen et al., 2016). Charged multivesicular body protein 4C(CHMP4C) belongs to the CHMP family. It encodes a protein that is part of ESCRT-III (endosomal sorting complex needed to transport III) which mediates membrane separation during cytoplasmic separation (Liu et al., 2021a). Some studies show that CHMP4C plays an important regulatory role in the tumor cell cycle (Li et al., 2015). Signal transducer and activator of transcription (STAT) is an important nuclear transcription factor. STAT4, as a member of its family, can affect immune cells and function by regulating the response of lymphocytes to IL-12 and regulating the differentiation of T cells. The abnormality of immune cells and function has been proved to be involved in immune escape and metastasis of tumor cells, thus regulating the occurrence and development of tumors (Cheng et al., 2020; Ebersbach et al., 2021). These four NRGs have been reported to be closely related to tumor development. Thus, we further explored the expression of the four NRGs in PDAC. Our detection results are basically consistent with the risk model. Only CHPM4C had low mRNA expression, not high mRNA expression, which was opposite to its protein expression. This result may be associated with post-translational modification, but its potential mechanism needs to be further explored and verified. In addition, whether the four genes regulate the development of PDAC through necroptosis also needs to be further explored *in vivo* and *in vitro*.

The molecular mechanism of necroptosis is a new hot spot in tumor research, but the relationship between tumor immunity and necroptosis is rarely reported. Previous studies have also shown that the presence of T cell infiltration predicts a better prognosis (Zuazo et al., 2020; Scott et al., 2021). Meanwhile, studies also have shown that chimeric antigen receptor T cell immunotherapy is an effective choice for the treatment of drug-resistant (Titov et al., 2021). In keeping with these studies, the results of ssGSEA analysis we performed in TCGA and GEO cohorts showed that the infiltration of CD8+T cells, helper T cells (Th), follicular helper T cells (Tfh) and Th1 cells in the low-risk group was significantly higher than that in the high-risk group. In addition, the activity of immune functions such as T cell costimulation and type II interferon response in the low-risk group was also significantly higher than that in the high-risk group. Type II interferon IFN- γ is mainly secreted by activated T cells, which have been reported to play an important role in the anti-tumor effect of pancreatic cancer (Kosmidis et al., 2018). These results indicated that our NRGs-based risk model was related to tumor immunity.

Although our study constructed an effective prognostic model for predicting the prognosis of PDAC patients, there are still some limitations. First of all, we need more clinical data and prospective studies are needed to verify the clinical effectiveness of this model. In addition, further cellular and animal experiments are needed to reveal the function and specific molecular mechanism of NRGs on the progression of PDAC. Finally, we have only done a preliminary theoretical study on the relationship between NRGs and immune status, and more basic experiments are also needed.

## Conclusion

In conclusion, we comprehensively and systematically analyzed the expression of NRGs in PDAC and constructed a novel risk prognostic based on four NRGs. This model could be used as an independent prognostic factor for PDAC and well predict the prognosis of PDAC patients.

## Data Availability

The original contributions presented in the study are included in the article/Supplementary Material, further inquiries can be directed to the corresponding authors.
