# Supplementary material for: Identification and Validation of Prognostic Model for Pancreatic Ductal Adenocarcinoma Based on Necroptosis-Related Genes
Source: Front Genet. 2022 Jun 16;13:919638. doi: 10.3389/fgene.2022.919638 (PMC9243220; doi:10.3389/fgene.2022.919638)
Supplement: Supplementary file 1 [file DataSheet2.PDF]

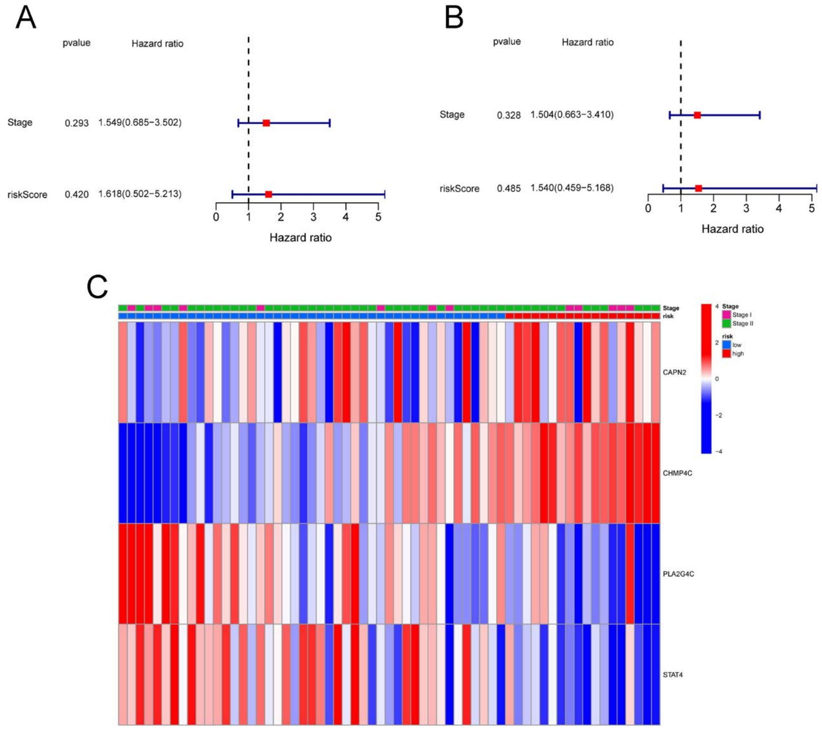

Supplementary Figure S2 | Univariate and multivariate Cox regression analyses for the risk score and clinical features in the GEO cohort. **(A)** Univariate Cox regression analysis. **(B)** Multivariate Cox regression analysis. **(C)** Heatmap between the risk score and clinical features.
