# Supplementary material for: Identification and Validation of Prognostic Model for Pancreatic Ductal Adenocarcinoma Based on Necroptosis-Related Genes
Source: Front Genet. 2022 Jun 16;13:919638. doi: 10.3389/fgene.2022.919638 (PMC9243220; doi:10.3389/fgene.2022.919638)
Supplement: Supplementary file 6 [file Table2.DOCX]

**Supplementary Table S2 |** Primers sequences used for qRT-PCR amplification.

| CAPN2 | Forward (5'‐3') | TCTTTGTGCATTCAGCCGAAG |
| --- | --- | --- |
|  | Reverse (5'‐3') | CTCATACCACTCAGCAATGCCTC |
| CHMP4C | Forward (5'‐3') | AGACTGAGGAGATGCTGGGCAA |
|  | Reverse (5'‐3') | TAGTGCCTGTAATGCAGCTCGC |
| PLA2G4C | Forward (5'‐3') | GGAAGACTGGTCAGAACTCACC |
|  | Reverse (5'‐3') | GCATTAGCAACAGCCCTTCTCC |
| STAT4 | Forward (5'‐3') | CTGCAAGACGAATTTGACTACAGG |
|  | Reverse (5'‐3') | GGCTGTTAAGCATTTCCTGCAG |
| GAPDH | Forward (5'‐3') | GTCTCCTCTGACTTCAACAGCG |
|  | Reverse (5'‐3') | ACCACCCTGTTGCTGTAGCCAA |
