# Supplementary figures and images for: Identification and Validation of Prognostic Model for Pancreatic Ductal Adenocarcinoma Based on Necroptosis-Related Genes
Source: Front Genet. 2022 Jun 16;13:919638. doi: 10.3389/fgene.2022.919638 (PMC9243220; doi:10.3389/fgene.2022.919638)

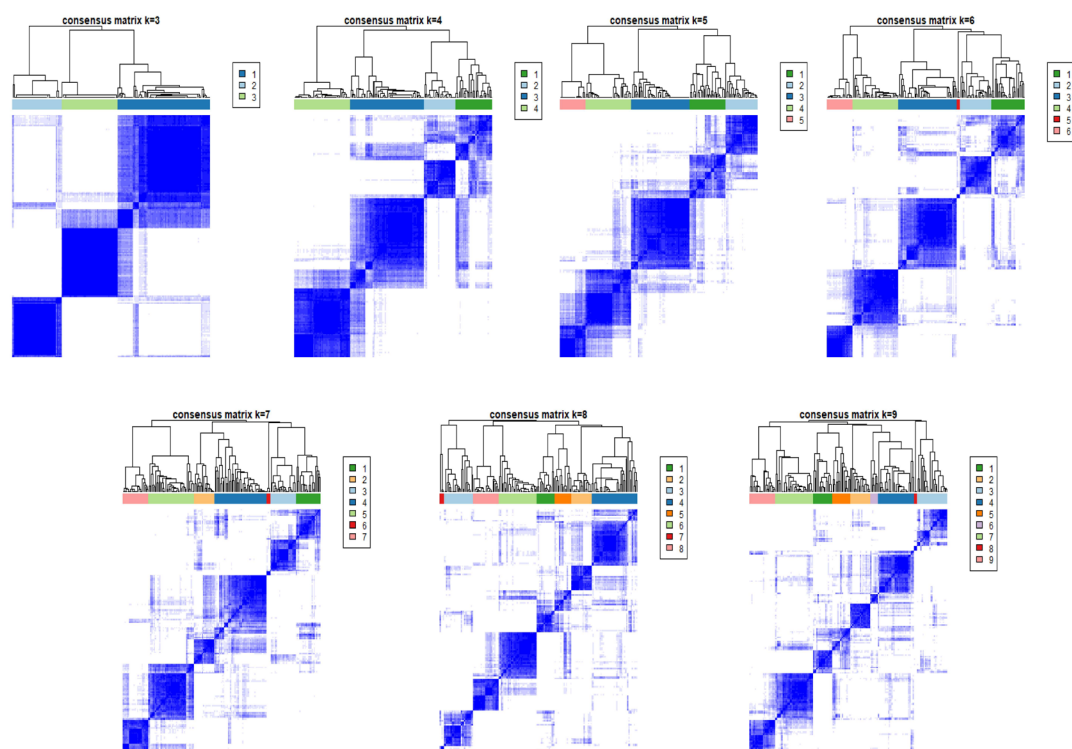

**Supplementary Figure S1** | The results of consensus clustering analysis ( $k$  from 3 to 9).

Supplement: Supplementary file 7 [file DataSheet1.PDF]
