# Supplementary material for: Identification and Validation of Prognostic Model for Pancreatic Ductal Adenocarcinoma Based on Necroptosis-Related Genes
Source: Front Genet. 2022 Jun 16;13:919638. doi: 10.3389/fgene.2022.919638 (PMC9243220; doi:10.3389/fgene.2022.919638)
Supplement: Supplementary file 8 [file Table3.DOCX]

| **Characteristics** | **TCGA** | **GSE57495** |
| --- | --- | --- |
| Number | 178 | 63 |
| Age, median (range) | 65(35-88) |  |
| Gender |  |  |
| Female | 80 (44.94%) |  |
| Male | 98 (55.06%) |  |
| Grade |  |  |
| G1-2 | 126 (70.79%) |  |
| G3-4 | 50 (28.09%) |  |
| unknow | 2 (1.12%) |  |
| TMN-stage |  |  |
| Stage I | 21 (11.79%) | 13 (20.63%) |
| Stage II | 146 (82.02%) | 50 (79.37%) |
| Stage III | 3 (1.69%) | 0 |
| Stage IV | 5 (2.81%) | 0 |
| unknow | 3 (1.69%) | 0 |
| T stage |  |  |
| T1-2 | 31 (17.42%) |  |
| T3-4 | 145 (81.46%) |  |
| unknow | 2 (1.12%) |  |
| N stage |  |  |
| Negative (N0) | 50 (28.09%) |  |
| Positive (N1-N2) | 123 (69.10%) |  |
| unknow | 5 (2.81%) |  |
| M stage |  |  |
| M0 | 79 (44.38%) |  |
| M1 | 5 (2.81%) |  |
| unknow | 94 (52.81%) |  |
| OS days (median) | 462.5 | 634 |
| Survival status |  |  |
| live | 86 (48.31%) | 21 (33.33%) |
| dead | 92 (51.69%) | 42 (66.67%) |

**Supplementary Table S3 |** The demographic and clinical characteristics of PDAC patients in different datasets.
